# Not Just the Demographic Change – The Impact of Trends in Risk Factor Prevalences on the Prediction of Future Cases of Myocardial Infarction

**DOI:** 10.1371/journal.pone.0131256

**Published:** 2015-07-27

**Authors:** Wolfgang Hoffmann, Jeanette Bahr, Kerstin Weitmann, Robert Herold, Thomas Kohlmann, Neeltje van den Berg

**Affiliations:** 1 Institute for Community Medicine, University Medicine Greifswald, Greifswald, Germany; 2 DZHK (German Centre for Cardiovascular Research), partner site Greifswald, Greifswald, Germany; Azienda Ospedaliero-Universitaria Careggi, ITALY

## Abstract

**Background:**

Previous predictions of population morbidity consider demographic changes only. To model future morbidity, however, changes in prevalences of risk factors should be considered. We calculated the number of incident cases of first myocardial infarction (MI) in Mecklenburg-Western Pomerania in 2017 considering the effects of demographic changes and trends in the prevalences of major risk factors simultaneously.

**Methods:**

Data basis of the analysis were two population-based cohorts of the German Study of Health in Pomerania (SHIP-baseline [1997–2001] and the 5-year follow-up and SHIP-Trend-baseline [2008–2011] respectively). SHIP-baseline data were used to calculate the initial coefficients for major risk factors for MI with a Poisson regression model. The dependent variable was the number of incident cases of MI between SHIP-baseline and SHIP-5-year follow-up. Explanatory variables were sex, age, a validated diagnosis of hypertension and/or diabetes, smoking, waist circumference (WC), increased blood levels of triglycerides (TG) and low-density-lipoprotein cholesterol (LDL), and low blood levels of high-density-lipoprotein cholesterol (HDL). Applying the coefficients determined for SHIP baseline to risk factor prevalences, derived from the new cohort SHIP-Trend together with population forecast data, we calculated the projected number of incident cases of MI in 2017.

**Results:**

Except for WC and smoking in females, prevalences of risk factors in SHIP-Trend-baseline were lower compared to SHIP-baseline. Based on demographic changes only, the calculated incidence of MI for 2017 compared to the reference year 2006 yields an increase of MI (males: +11.5%, females: +8.0%). However, a decrease of MI (males: -23.7%, females: -17.1%) is shown considering the changes in the prevalences of risk factors in the projection.

**Conclusions:**

The predicted number of incident cases of MI shows large differences between models with and without considering changes in the prevalences of major risk factors. Hence, the prediction of incident MI should preferably not only be based on demographic changes.

## Introduction

In Germany, ongoing demographic changes will influence the age-associated morbidity in the population over the next decades [1, 2]. The expected relative as well as absolute increase of patient numbers will affect the medical infrastructure and will challenge future provision of adequate diagnostics, treatment, and care [3, 4, 5, 6]. Valid predictions of changes in morbidity of age-associated chronic diseases e.g. cardiovascular and metabolic disease or cancer are important for planning future health services delivery. Diseases of the cardiovascular system such as coronary heart disease are very common in industrial countries [7]. Myocardial infarction (MI) is a frequent acute complication of coronary heart disease. Model based scoring systems have been developed to calculate the risk of coronary events [8, 9, 10]. Assmann et al. present a point-scoring scheme for calculating the risk of an acute coronary event (fatal or nonfatal myocardial infarction or acute coronary death) using the Cox proportional hazards model in conjunction with survival curves and the categories of selected risk factors observed in epidemiologic studies [8]. These scores allow for individual risk estimation and should trigger preventive measures. Hence, future numbers of incident cases will be a net effect of the increasing proportion of the elderly in the population and the prevalences of major risk factors which are also likely to change over time.

The aim of this work was to determine the absolute number of patients with first incidence of MI in the German Federal State of Mecklenburg-Western Pomerania for the year 2017 as a quantitative basis for future demands of medical care. Previous prognoses of morbidity focused only on the effect of the demographic changes, implicitly assuming all other influencing factors to be constant over time. More realistic models of future morbidity, however, should consider not only the changing demography, but also trends in the prevalences of major risk factors. We calculated the number of incident cases of first MI in 2017 modelling simultaneously 1) changes in total population numbers, age and sex distribution, and 2) trends in major risk factor prevalences.

## Methods

### SHIP and SHIP-Trend

MI-incidence numbers and risk factor prevalences were derived from two population-based epidemiological cohorts within the Study of Health in Pomerania (SHIP and SHIP-Trend), both conducted in the German region of Western Pomerania. For both cohorts, stratified samples were drawn from the total population of Western Pomerania comprising about 213 000 inhabitants in 1996. Stratification variables were age, sex, and place of residence. The baseline examination of SHIP (N = 4,308, response: 68.8%) was performed between 1997 and 2001, the 5-year follow-up in this cohort (N = 3,300, response: 83.6%) between 2002 and 2006.

Between 2008 and 2011, the baseline examination for a newly drawn random sample of participants was conducted (SHIP-Trend, N = 4,248, response: 50.0%). This sample was retrieved from the same study area, but completely independent from the SHIP population [11, 12].

### Ethics statement

Written informed consent was obtained from all study participants. Both cohort studies were approved by the ethics committee of the University Medicine Greifswald.

### Risk factors

The risk factors, included in the analysis, were selected on the basis of the results of the population based PROCAM study in which 9 risk factors for MI were identified: gender, age, physician diagnosis of hypertension, physician diagnosis of diabetes, smoking, obesity (operationalized as waist circumference (WC) ≥ 94 cm in males and ≥ 80 cm in females), high levels of triglycerides (TG) (value of > 1.7 mmol/l), low high density lipoprotein cholesterol (HDL) (value < 1 mmol/l in males and <1.2 mmol/l in females) and high levels of low density lipoprotein cholesterol (LDL) (value of > 3.0 mmol/l) [8]. The used cut off limits were retrieved from the literature [13,14,15]. Hypertension and diabetes were based on self-reported physicians’ diagnoses or self-reported disease-specific medication as assessed in the standardized personal face-to-face interview (CAPI). Smokers were categorized into current, former, and never smokers. Participants who smoked at least 1 cigarette per day during the last five years were considered current smokers. Waist circumference was measured between the lower rib margin and the iliac crest in the horizontal plane.

TG concentration was determined enzymatically using reagents from Nobis (Fa.Nobis, Endingen; Germany) on Hitachi 717 (Roche Diagnostics, Mannheim, Germany) in SHIP. HDL and LDL were measured on Epos 5060 (Eppendorf, Hamburg, Germany) using phosphotungstate/MgCl_2_ and dextran sulphate precipitation (Immuno, Heidelberg, Germany) in SHIP. In SHIP-Trend, the metabolites of TG, HDL, and LDL were measured using Dimension Vista reagents on Siemens VISTA (Siemens Healthcare Diagnostics, Eschborn, Germany). The SHIP laboratories take part in the official German external quality proficiency testing programmes [12].

Data on the prevalences of hypertension, diabetes, smoking, obesity, high TG, low HDL and high LDL were retrieved from the SHIP baseline and the SHIP-Trend baseline examination. Participants with a previous MI (self-reported physicians’ diagnosis or self-reported disease-specific medication), unknown history of MI or missing data with respect to risk factors as well as participants older than 80 years (due to an insufficient number of participants) were excluded. Participants who participated both in the baseline examination and the 5-year follow-up of SHIP, as well as those, who attended the baseline examination but died before 5-year follow-up with a known cause of death, were included in the analysis. Cause of death was retrieved from death certificates provide by the health authorities. Between SHIP baseline and the 5-year follow-up an additional morbidity follow-up was performed by postal questionnaires or telephone interviews.

Participants with no history of MI at the time of the SHIP baseline examination but (1) with a documented, self-reported MI in the time interval between SHIP baseline and the 5-year follow-up or (2) with one of the following ICD-10 codes as the cause of death: I21.9, I24.9, I25.0, I25.1, I25.9, I28.8 in the same period were defined as incident cases of MI [16].

We defined 2006 as the base year for the projection of the prognosis (end of SHIP 5-year follow-up examination). General population data by 5-year age group and sex for 2006 were provided by the German Federal Statistical Office [17]. Population forecasts for the year 2017 (projected end of the 5-year follow-up examination of SHIP-Trend) were provided by the Statistical Office of Mecklenburg-Western Pomerania [18].

### Statistical analysis

**Fig 1:**
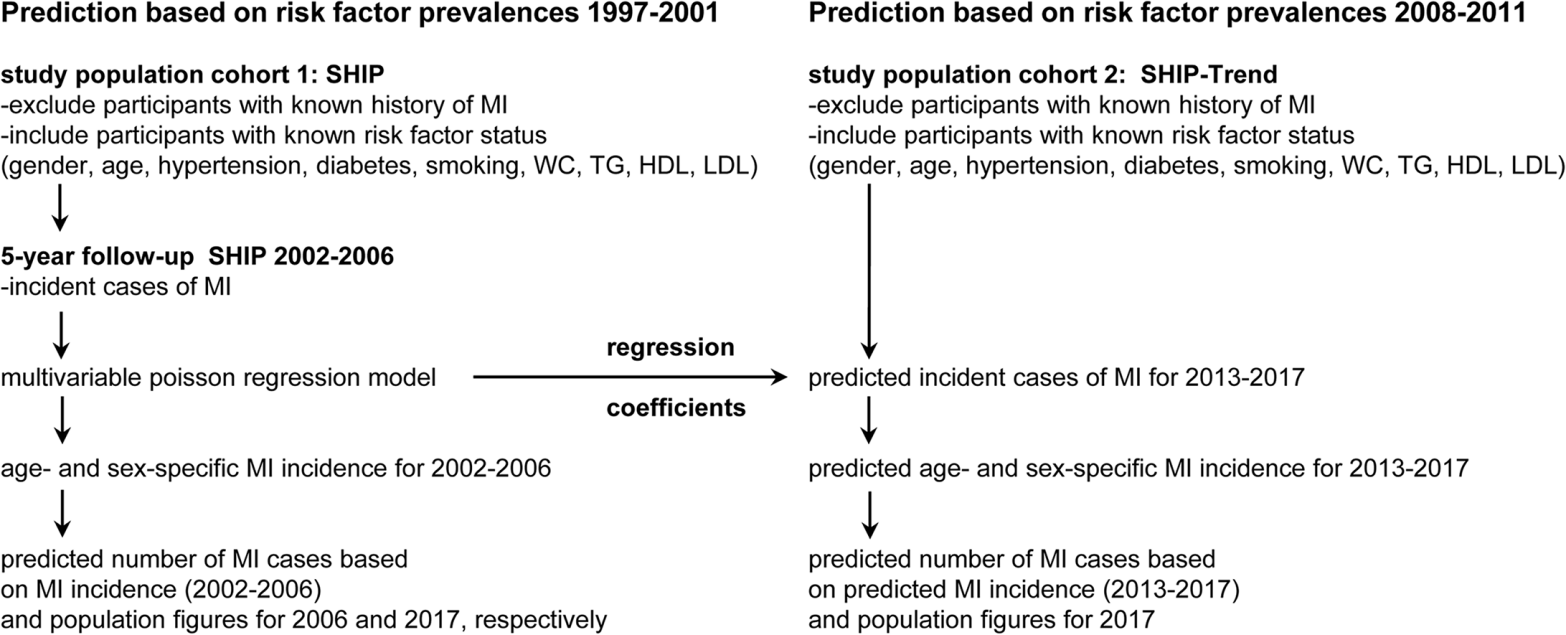
Flow diagram of the analysis strategy (Abbreviations: WC, waist circumference; TG, triglyceride; HDL, high-density-lipoprotein; LDL, low-density-lipoprotein).

A Poisson regression was used to fit the models. As dependent variable we used the number of incident cases of MI in the period between SHIP baseline and the 5-year follow-up. Explanatory variables were risk factors including sex, age, diagnosed hypertension, diagnosed diabetes, smoking, WC, TG, LDL, and HDL. Age-group and sex specific prevalences of the risk factors were calculated based on SHIP-baseline and used to calculate the coefficients for each of the risk factors for incident MI. In the next step, the coefficients of the Poisson regression model determined for the risk factor prevalences in SHIP-baseline were used to compute the unknown MI counts for the 5-year period following the baseline examination of SHIP-Trend (prediction model). Based on the resulting counts, we computed the 1-year incidence of MI for the SHIP-Trend sample separately for 12 age-groups (20–24, 25–29, 30–34, 35–39, 40–44, 45–49, 50–54, 55–59, 60–64, 65–69, 70–74, and 75–79) and for both genders. The annual number of incident cases of MI was assumed constant for all ages within the same 5-year age group. Using the population data, we projected the number of incident cases of MI for Mecklenburg-Western Pomerania for 2006 and 2017 respectively. For the projection of incident cases of MI in 2006 and 2017, confidence intervals were constructed by calculating asymptotic confidence intervals. The flow diagram of this projection is shown in Fig 1.

To allow for comparison, the risk factor prevalences derived from SHIP and SHIP-Trend, respectively, were age- and sex-standardized to the population from Mecklenburg-Western Pomerania in 2006 using the direct method of standardization.

Descriptive results are provided as mean ± the standard deviation (SD) or frequency. Statistical analyses were performed using R 3.0.0, a public domain software package for statistical computing and the generation of graphics.

Summarized, the projection consisted of the following steps:

1. The risk factor prevalences based on data of SHIP baseline and the incidence of MI between SHIP baseline and 5-year follow-up were used to fit a multivariable Poisson model to estimate the coefficient for each risk factor (Fig 1, left column).
2. Based on this multivariable model, age- and sex-specific MI-incidence were calculated. To obtain the number of MI-cases in Mecklenburg-Western Pomerania, these incidences were applied to each age and sex group of the population of Mecklenburg-Western Pomerania for 2006 and 2017, respectively (Fig 1, left column. In this projection, we assumed that risk factor prevalences remain constant between 2006 und 2017.
3. Using the baseline examination of SHIP Trend allowed us to calculate the cases of MI for the 5-year period following the baseline examination of SHIP-Trend, based on the risk factors prevalences measured in SHIP Trend. Keeping the coefficients of the multivariable Poisson model and plugging in the SHIP Trend risk factor prevalences, we obtained the numbers of cases of MI per age and sex group. These numbers were again projected to the population in Mecklenburg-Western Pomerania for 2017 (Fig 1, right column).

## Results

**Fig 2:**
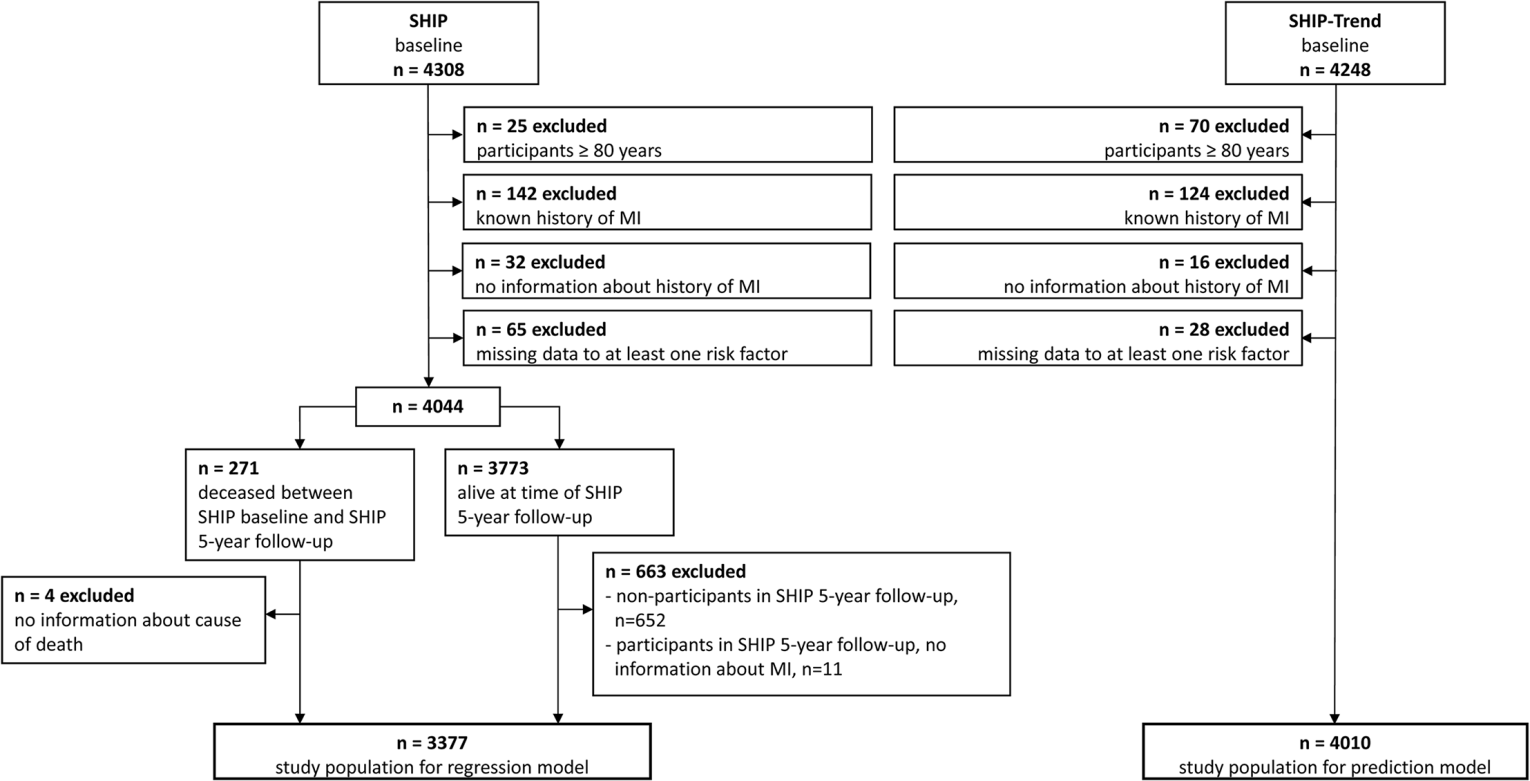
Flow diagram of the data processing.

Fig 2 depicts the generation of the analysis data set for each of the two cohorts. 3,377 (male: n = 1632, female: n = 1745) participants of SHIP and 4,010 (male: n = 1905, female: n = 2104) participants of SHIP-Trend were included respectively.

### Risk factors

Table 1 shows the unadjusted basic characteristics of each risk factor from the baseline examinations of SHIP and SHIP-Trend. The prevalences of most risk factors were lower in SHIP-Trend compared to SHIP.

**Table 1:** Means and prevalences (unadjusted) of the risk factors from the baseline examinations of SHIP and SHIP-Trend.

	males		females	
risk factor^#^	SHIP	SHIP-Trend	SHIP	SHIP-Trend
	n = 1632	n = 1906	n = 1745	n = 2104
age (year)	50.93 ± 15.79	51.34 ± 15.15	49.01 ± 15.54	50.6 ± 14.82
hypertension (%)	63.60	48.69	42.06	39.12
diabetes (%)	12.44	10.49	8.77	8.37
smoking (%)	32.05	29.49	24.93	24.86
WC (cm)	95.86 ± 11.5	97.09 ± 12.90	82.94 ± 12.86	85.02 ± 13.58
proportion WC ≥ 94cm (%)	55.64	57.35		
proportion WC ≥ 80cm (%)			54.84	58.65
TG (mmol/l)	2.11 ± 1.42	1.90 ± 1.53	1.56 ± 1.18	1.44 ± 0.83
proportion TG > 1.7mmol/l (%)	50.18	43.44	30.95	26.57
HDL (mmol/l)	1.30 ± 0.37	1.28 ± 0.33	1.60 ± 0.43	1.59 ± 0.37
proportion HDL < 1.0mmol/l (%)	19.36	17.16		
proportion HDL < 1.3mmol/l (%)			16.10	14.02
LDL (mmol/l)	3.68 ± 1.16	3.36 ± 0.94	3.53 ± 1.18	3.38 ± 0.98
proportion LDL > 3.0mmol/l (%)	72.12	63.48	63.50	62.36

^#^Abbreviations: WC, waist circumference; TG, triglyceride; HDL, high-density-lipoprotein; LDL low-density-lipoprotein

Definition hypertension or diabetes: self-reported physicians’ diagnosis or self-reported specific medication

Definition smoking: Participants who smoked at least 1 cigarette per day during the last five years were considered current smokers

**Fig 3:**
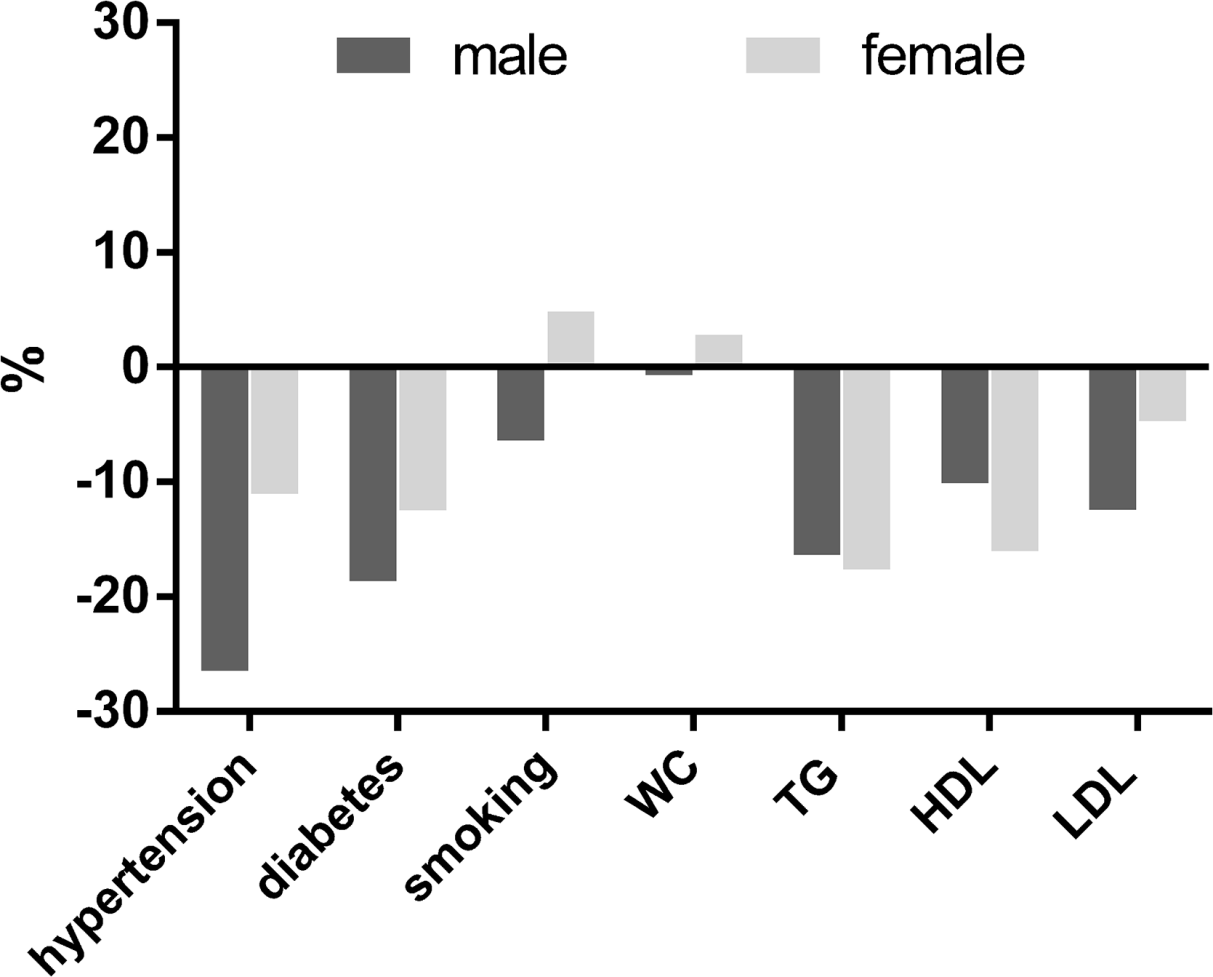
Relative changes of the prevalences of risk factors from SHIP-Trend compared to SHIP prevalences, age- and sex-standardized on the population of from Mecklenburg-Western Pomerania in 2006. Abbreviations: WC, waist circumference; TG, triglyceride; HDL, high-density-lipoprotein; LDL, low-density-lipoprotein.

Risk factor prevalences were age-standardised to allow for a direct comparison between SHIP and SHIP-Trend and to determine the relative changes of the prevalences (Fig 3). All risk factors decreased in SHIP-Trend compared to SHIP except WC and smoking in females which show slight increases. The changes show a sex-specific pattern: the decreases in hypertension, diabetes, and LDL are more pronounced in males compared to females, whereas favourable changes in low HDL are more pronounced in females.

### Projection of incident cases of MI in 2017

In SHIP, 67 cases (males: n = 42, females: n = 25) of first MI were observed between the baseline examination and the 5-year follow up. Of these, 33 were abstracted from death certificates. 18 were coded with I21.9 (acute myocardial infarction, unspecified), 10 with I25.9 (chronic ischaemic heart disease) as the primary cause of death. Since chronic ischaemic heart disease is not the actual cause of death but rather the underlying disease, we assumed an imprecision in the coding by the physician who certified the death. In these cases, we imputed a myocardial infarction as the most plausible cause of death.

**Fig 4:**
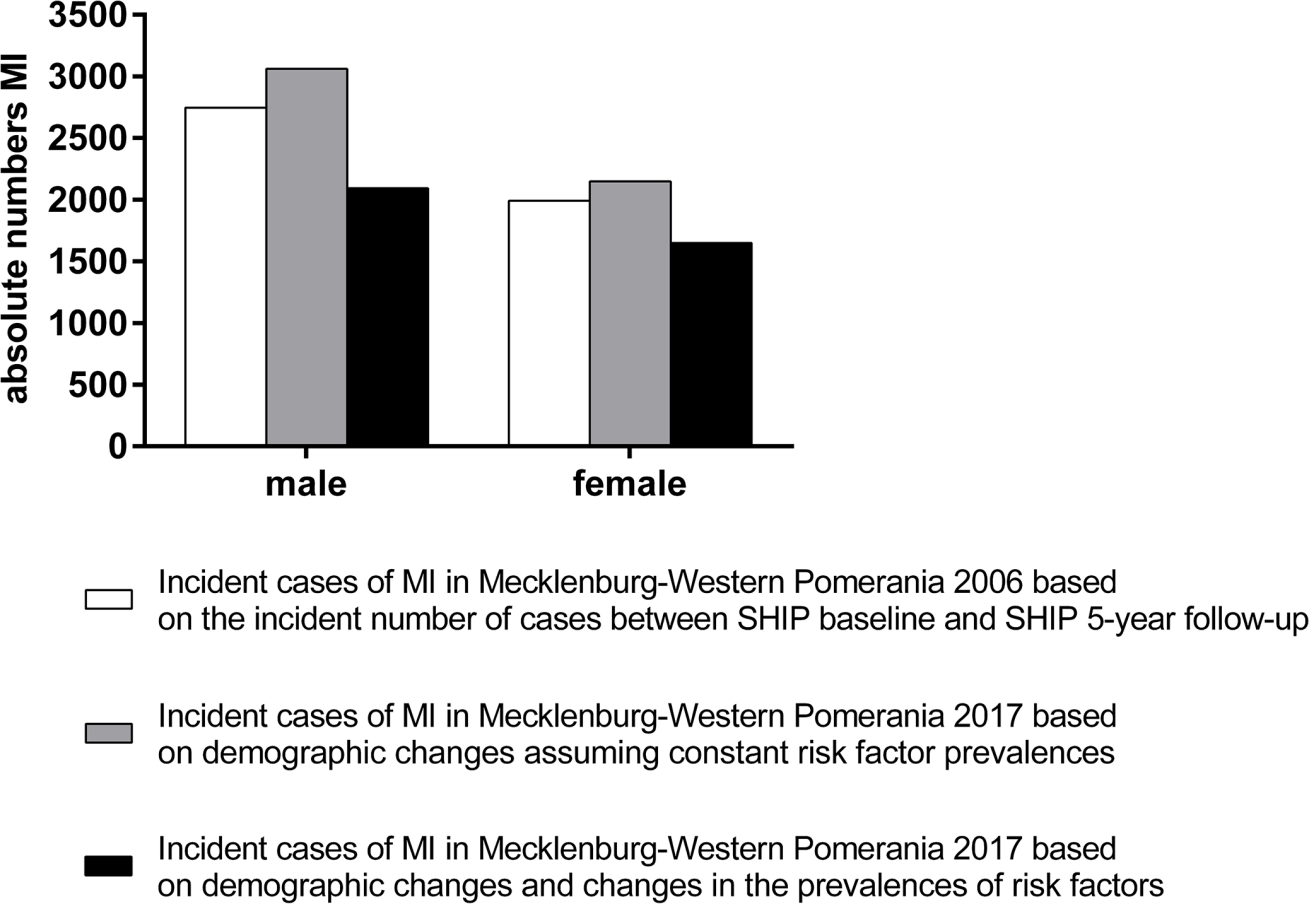
Absolute numbers of incident cases of MI in Mecklenburg-Western Pomerania in 2006 and 2017 with and without considering the changes in the prevalences of risk factors between 1997–2001 and 2008–2011.

Assuming that the age- and sex-specific prevalences of risk factors as determined in SHIP (Table 2) remain constant, the demographic changes in age and sex distribution of the population will cause an increase in the number of incident cases in Mecklenburg-Western Pomerania of 11.5% in males (2006: 2745 incident cases (95% CI: 2531–2957); 2017: 3061 incident cases (95% CI: 2830–3289); +316 cases) and 8.0% in females (2006: 1990 incident cases (95% CI: 1851–2369); 2017: 2149 (95% CI: 1954–2436); +159 cases) between 2006 (white bars in Fig 4) and 2017 (grey bars in Fig 4).

**Table 2:** Results of the Poisson regression model (dependent variable: MI incidence between SHIP baseline and 5-year-follow-up. The coefficients of the risk factors of this model were used to compute the MI counts for the 5-year period following the baseline examination of SHIP-Trend.

Risk factor	Coefficient	95% CI	Standard Error	p-value
**Sex**	-0,123	-0,688–0,442	0,288	0,670
**Age**	0,069	0,044–0,093	0,012	0,000
**Hypertension**	1,232	0,351–2,112	0,449	0,006
**Diabetes**	0,507	-0,037–1,051	0,277	0,068
**Smoking**	0,678	0,098–1,258	0,296	0,022
**Waist circumference**	0,007	-0,016–0,030	0,012	0,539
**Triglyceride**	-0,092	-0,299–0,114	0,105	0,382
**High-density-lipoprotein**	-0,726	-1,511–0,059	0,400	0,070
**Low-density-lipoprotein**	0,293	0,109–0,478	0,094	0,002

CI: Confidence Interval

Applying the estimated regression coefficients to the risk factor prevalences determined in SHIP-Trend the numbers of cases of MI in both males and females would decrease in 2017 compared to 2006 (2094 (95% CI: 2016–2346) vs. 2745 cases (-23,7%) and 1649 (95% CI: 1502–1827) vs. 1990 cases (-17,1%), in males and females, respectively (black bars in Fig 4).

## Discussion

In this study, two population based cohorts were used to examine the effects of changing prevalences of risk factors on the prediction of future numbers of cases of first MI in Mecklenburg-Western Pomerania. In the first scenario, incident cases of MI were calculated including demographic changes only, assuming constant risk factor prevalences over time. According to this scenario the number of incident cases of MI will rise in 2017 compared to the year 2006 as a consequence of the demographic change with an increasing absolute number of people in the age groups with higher incidences of MI. Projected case numbers for other age-related diseases including stroke, cancer and neurodegenerative diseases show similar tendencies [2, 13].

The second scenario considers the changes in the prevalences of risk factors between SHIP baseline and SHIP-Trend baseline. Somewhat unexpected, this scenario yields a decrease of the number of cases with incident MI in 2017 compared to 2006. This pattern shows the considerable impact of other risk factors than just age and sex on MI incidence. In fact, the decline in the prevalence of several of the major risk factors over the approximately 10 year period between 1997–2001 and 2008–2011 more than offsets the effect of the demographic change.

These predictions are obviously limited by a variety of uncertainties, including high risk groups that could be underrepresented in the cohort population, or positive effects of earlier diagnosis and better treatment of risk factors such as diabetes or hypertension over time. Likewise, we could not consider any psychosocial and societal change over time which could considerably affect incidence of MI.

A number of other risk factors that are well established in the literature could not be considered in our models. Occupational status and nutrition influence cardiovascular events [14,14,19] as well as genetic aspects [20, 21]. Only waist circumference was used in our modelling while waist-to-height ratio has been suggested as a better predictor of cardiovascular risk and mortality [22]. The risk factors hypertension and diabetes and incident MI were estimated based on self-reported physician diagnoses. This causes some uncertainty in the validity of the diagnoses. Incident cases of MI in the population are underestimated because people aged 80 and older could not be included in the analysis.

A major strength of this analysis is that risk factor prevalences have been measured in two population-based epidemiologic cohort studies adopting highly standardized examinations and laboratory protocols, extensive quality management, and almost complete follow-up.

In Germany, data on trends in MI morbidity from the population-based MONICA/KORA registry of acute myocardial infarction covering 18 years of registration, confirm decreasing MI morbidity and mortality over time [23]. Corresponding trends can be observed in other countries and have been explained by a decrease in the prevalence of risk factors as well as by improved prevention measures and better medical care [24, 25, 26].

Concluding, our modelling should caution against any oversimplistic projection of case numbers of age-related diseases based only on demographic changes in a population. These projections assume, often tacitly, that the prevalences of risk factors in a population remain constant over time. Our results show, that this is not necessarily the case. As a consequence, predictions should be adjusted for changes in prevalence of major risk factors to more realistically reflect time trends in epidemiology and healthcare research.
